# Clinical and Pathogenetic Notes

**Published:** 1891-05

**Authors:** E. W. Berridge

**Affiliations:** London, England


### CLINICAL AND PATHOGENIC NOTES.

E. W. Berridge, M. D., London, England.

Nux Vomica.—I gave Mrs. F. one dose of *Nux-vomica*$^{102m}$ (F. C.) for uterine troubles. She woke next morning about six A. M. with umbilical colic, continuing all day, radiating from umbilicus across abdomen, up to right mamma, and through to angle of right scapula.

Lycopodium.—Mrs. —— wrote: "At four P. M., for two days, I have been feeling rather sick at stomach, giddy, and pains down back of head, coming from the top; I feel the pain as it were in a stripe of an inch wide; feel cold, no appetite, very depressed, and wake very early in the morning. To-day have a sharp attack of rheumatism in right shoulder and a sharp, but not lasting attack in foot and ankle. I feel done up."

One dose of *Lycopodium*$^{mm}$ (H. S.) cured.

Carbo-vegetabilis.—Dr. Fourness Simmons, of Brisbane, Australia, wrote out for me the following effects of using charcoal tooth-powder:

The best selected remedies relieved only temporarily till the patient left off the exciting cause.

Epistaxis after blowing nose; worse on warm days. Feeling of weakness in both upper eyelids. Sensation of pain of sand in both inner canthi. Deep pulsating pains in both eyeballs, shortly after reading. Long-sightedness. Seminal emissions, without dreams, frequently repeated; followed next morning by headache and pain in back. Erections in morning, rousing from sleep at three or four A. M., with strong desire to urinate, though he passed but little. Sleeplessness after three or four A. M. Frequent hawking of thick mucus. Cannot sleep at night unless lying first on right side, then on the left, and finally turning on right side.

He never had these symptoms before, and they lingered on for more than four years later.

Mercurius Solubilis.—Mr. —— wrote from Liverpool,

August 21st, 1889: "I have found out within the last ten days that a hard chancre has appeared on my prepuce, and the injured glands are slightly swollen. No other symptoms, except that the legs feel a little tired.

"Dr. —— (allopath) examined me and gave me the following prescription: A pill of *Mercury with Chalk* and *Dover's Powder* twice daily, and a lotion of *Lead and Opium*. As I do not care to take allopathic doses I am at present, by the advice of Dr. —— [a mongrel pretending homœpathy], using *black wash* as a lotion and *Nitric acid* as medicine. My brother had such confidence in you and your medicines that he told me that if ever I got anything the matter to write to you; and I think you will agree when I say I have contracted about the worst disease on earth."

I sent on August 22d one dose of *Mercurius-solubilis*$^{6\,cm}$ (Fincke) and instructed him to leave off all quackish mongrel treatment and simply apply wet lint to the chancre. On August 24th he wrote: "After taking the powder I felt myself again, all the symptoms leaving me, except the swellings in the groin, but they are no larger than peas. The chancre is healing up very quickly." No further report.

Phosphoric Acid.—November 3d, 1888, I gave a patient *Phosph-acid*$^{cm}$ (F. C.) a daily dose for seven days. On November 15th he reported that he had "feeling in stomach as if everything had stuck fast and was dry." He says the *Phosphoric acid* always causes this symptom with him.

